# Engineering of a high lipid producing *Yarrowia lipolytica* strain

**DOI:** 10.1186/s13068-016-0492-3

**Published:** 2016-03-31

**Authors:** Jonathan Friedlander, Vasiliki Tsakraklides, Annapurna Kamineni, Emily H. Greenhagen, Andrew L. Consiglio, Kyle MacEwen, Donald V. Crabtree, Jonathan Afshar, Rebecca L. Nugent, Maureen A. Hamilton, A. Joe Shaw, Colin R. South, Gregory Stephanopoulos, Elena E. Brevnova

**Affiliations:** Total New Energies, 5858 Horton Street, Emeryville, CA 94610 USA; Novogy, Inc., 85 Bolton Street, Cambridge, MA 02140 USA; Department of Chemical Engineering, Massachusetts Institute of Technology, 77 Massachusetts Ave., Cambridge, MA 02139 USA; Twist Bioscience, 455 Mission Bay Blvd South, San Francisco, CA 94158 USA; Evelo Therapeutics, 620 Memorial Dr., Cambridge, MA 02139 USA

**Keywords:** *Yarrowia lipolytica*, Lipid accumulation, Oleaginous yeast, Metabolic engineering

## Abstract

**Background:**

Microbial lipids are produced by many oleaginous organisms including the well-characterized yeast *Yarrowia lipolytica*, which can be engineered for increased lipid yield by up-regulation of the lipid biosynthetic pathway and down-regulation or deletion of competing pathways.

**Results:**

We describe a strain engineering strategy centered on diacylglycerol acyltransferase (DGA) gene overexpression that applied combinatorial screening of overexpression and deletion genetic targets to construct a high lipid producing yeast biocatalyst. The resulting strain, NS432, combines overexpression of a heterologous DGA1 enzyme from *Rhodosporidium toruloides*, a heterlogous DGA2 enzyme from *Claviceps purpurea*, and deletion of the native TGL3 lipase regulator. These three genetic modifications, selected for their effect on lipid production, enabled a 77 % lipid content and 0.21 g lipid per g glucose yield in batch fermentation. In fed-batch glucose fermentation NS432 produced 85 g/L lipid at a productivity of 0.73 g/L/h.

**Conclusions:**

The yields, productivities, and titers reported in this study may further support the applied goal of cost-effective, large -scale microbial lipid production for use as biofuels and biochemicals.

**Electronic supplementary material:**

The online version of this article (doi:10.1186/s13068-016-0492-3) contains supplementary material, which is available to authorized users.

## Background

Microbial lipid production has received applied interest both for the synthesis of specialty chemicals and for the production of fuels and bulk chemicals from low-cost carbon feedstocks [1]. Considering the high demand for lipid molecules, augmentation of current plant- and animal-based production by microbial conversion of starch and sugar crops, agricultural residues, and lignocellulosic material has been the subject of many recent studies. Among natively oleaginous organisms, which are capable of producing lipids at a level greater than 20 % of their dry cell weight, the well-characterized yeast *Yarrowia lipolytica* [2] has received particular interest. Wild-type *Y. lipolytica* can accumulate lipids up to 36 % of dry cell weight from glucose [3] and 50–60 % of dry cell weight when fed exogenous fatty acid substrates for biomodification to higher value fats and oils [4, 5], and researchers have metabolically engineered strains to produce high value lipids [6–8]. In addition to production and bio-modification of lipids, *Y. lipolytica* strains are capable of secreting low-molecular weight secondary metabolites such as citric acid and polyols, and extracellular enzymes under specific fermentation conditions [9, 10]. Extensive genetic systems have been developed to manipulate this yeast [9, 11, 12].

Lipids serve as energy storage molecules and building blocks for cellular membranes and are composed of fatty acids, isoprenoids, and other water-insoluble hydrocarbon molecules. In many oleaginous yeasts, fatty acids are the dominant lipid compound, constituting 90–95 % of total lipid material [13, 14]. To avoid the potentially toxic and membrane-disturbing effects of free fatty acids, they are incorporated into nonpolar lipids such as triacylglycerols (TAG) and sterol esters. TAGs are stored in subcellular compartments termed lipid droplets or lipid bodies [15–18].

**Fig. 1:**
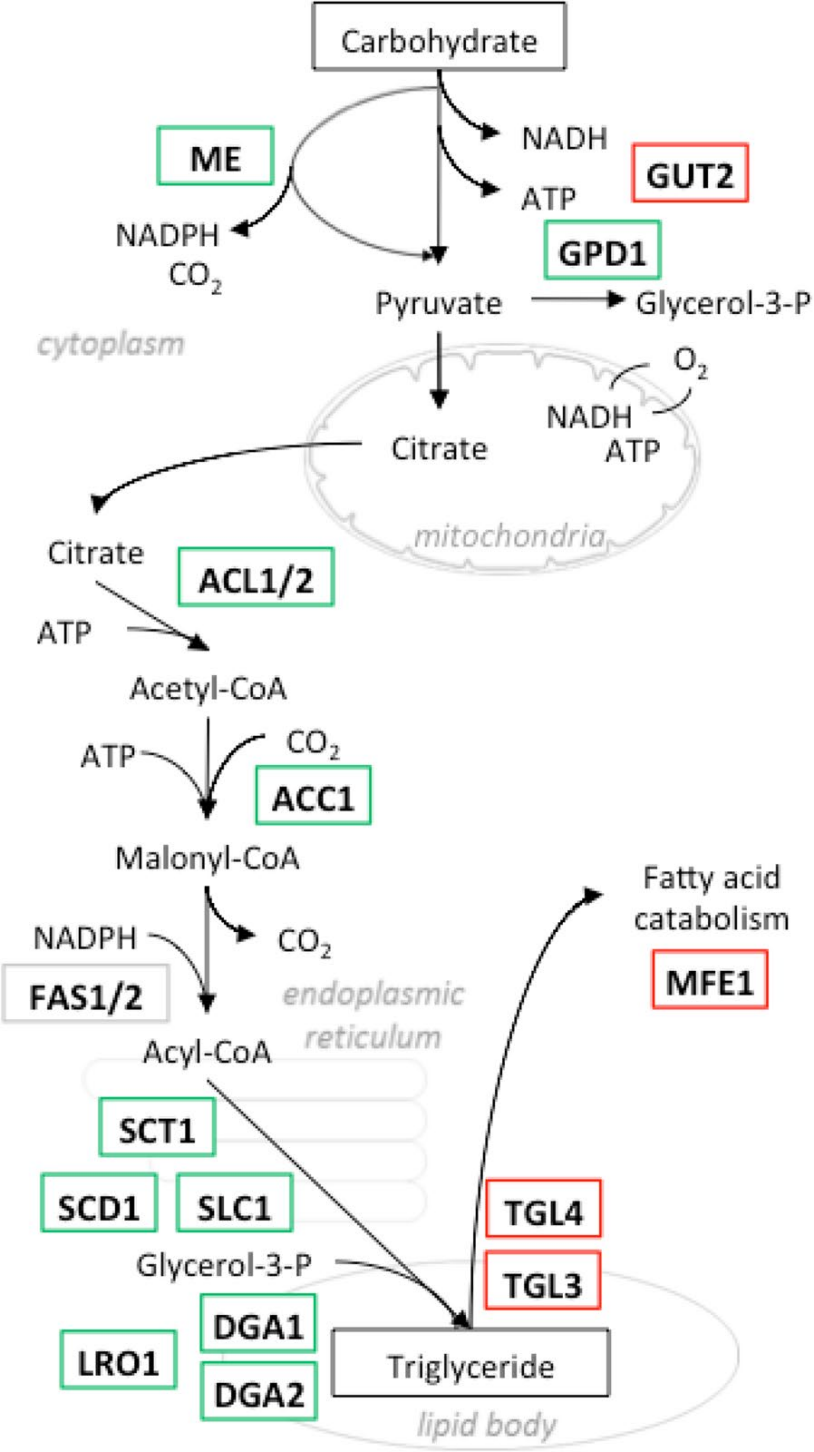
Major steps of lipid biosynthesis in *Y. lipolytica.* Overexpression genetic targets screened in this study for their effect on lipid production are shown in *green* *boxes*. Deletion genetic targets are shown in *red boxes*

Several applied studies have focused on increasing TAG production through genetic engineering of TAG synthesis and degradation pathways [19–21], including recent publications that have pushed previous limits of *Y. lipolytica* lipid productivity and titer [22–24]. A series of proteins were postulated as regulators of the conversion of carbohydrate sources into TAGs: malic enzyme (ME), ATP citrate lyase (ACL), Acetyl-CoA carboxylase (ACC), Bifunctional glycerol-3-phosphate/glycerone-phosphate O-acyltransferase (SCT), 1-acylglycerol-3-phosphate O-acyltransferase (SLC1), Stearoyl-CoA desaturase (SCD), and phospholipid: diacylglycerol Acyltransferase (LRO) (Fig. 1).

In particular, the enzymes DGA1 (diacylglycerol acyltransferase type 2) and DGA2 (diacylglycerol acyltransferase type 1) have been recognized as key components of the lipid pathway, performing the final step of TAG synthesis, incorporation of the third acyl-CoA onto the diacylglycerol backbone and transport into the lipid droplet [23]. Up-regulation of the native *Y. lipolytica* DGA1 and DGA2 enzymes have significantly increased lipid yield and productivity [19, 21, 26], and it has been hypothesized that efficient diacylglycerol acyltransferase activity creates a critical driving force for high levels of lipid accumulation in oleaginous organisms due to its dual role in TAG biosynthesis and transport into the lipid droplet [19, 27, 28]. Additionally, it has been shown that the deletion of DGA2 impairs TAG synthesis and lipid production in *Y. lipolytica* [25]. It has also been demonstrated that DGA1 or DGA2 overexpression is beneficial for lipid accumulation in *R. toruloides* [29] as well as in plant seeds [30].

In this study, we describe a systematic lipid biosynthesis engineering approach centered on diacylglycerol acyltransferase gene overexpression. First, we evaluated combinations of endogenous gene targets and then we optimized key enzymes by screening heterologous genes for engineering of an improved lipid-accumulating biocatalyst. The resulting strain of this process reached a 77 % lipid content and a 0.21 g lipid per g glucose yield in batch glucose fermentation, while in fed-batch glucose fermentation it produced 85 g/L lipid at a 0.73 g/L/h productivity.

## Results/discussion

### Overexpression of endogenous DGA1 in *Yarrowia lipolytica*

In an effort to develop a biocatalyst for industrial lipid production, we evaluated several *Y. lipolytica* wild-type strains for desirable biocatalyst qualities, such as minimal citric acid secretion, non-hyphal morphology, and ease of genetic manipulation. We isolated one such strain, designated NS18 (obtained from NRRL # YB-392), for future strain engineering. Previous studies have identified several endogenous genes which improve lipid production when overexpressed or deleted in *Y. lipolytica* [19, 21, 25, 31]. One of these genes, DGA1, encoding for diacylglycerol acyltransferase type 2, is involved in the final step of TAG synthesis [2, 19, 21, 25, 32–36] and has been a key genetic up-regulation target to increase production of TAG from glucose in independent *Y. lipolytica* studies [19, 21].

**Fig. 2:**
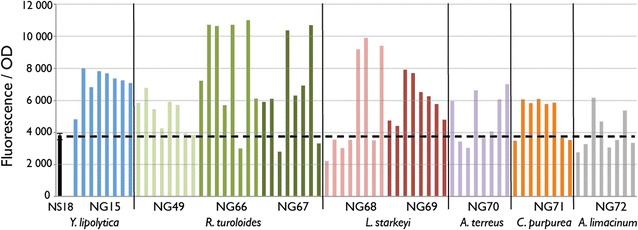
Overexpression of *DGA1* genes in *Y. lipolytica* strain NS18. Nine *DGA1* genes (Table 2) under the control of the *Y. lipolytica* GPD1 promoter were randomly integrated into the NS18 genome and 8 transformants for each gene were analyzed by fluorescence-based lipid assay after 72 h of growth in nitrogen-limited media. The average with standard deviation from triplicate experiments is shown for the parent strain. Fluorescence was measured at excitation 486 nm and emission 510 nm and normalized by cell optical density (OD) at 600 nm

In order to evaluate the effect of DGA1 overexpression in NS18, the *Y. lipolyticaDGA1* gene (NG15) was introduced into the NS18 genome under the control of the strong constitutive GPD1 promoter to produce strain NS297. Although use of promoters with varied expression levels may allow for a higher degree of metabolic balancing and control, we felt strongly constitutive promoters were suitable for identifying targets for lipid biosynthesis overexpression. To produce engineered strains, 50–200 transformants were screened in 96- or 48-well plates and evaluated by fluorescence-based assay for lipid content. The top isolates were then tested in 50-mL shake flasks and the highest performing strains were grown in 1 L bioreactors using a high cell density fed-batch glucose fermentation process followed by gas chromotograph (GC) lipid content analysis. Lipid content measured by GC is reported as fatty acid methyl ester equivalents, as described in the methods section. NS297 transformants exhibited a twofold increase in lipid content compared to wild-type *Y. lipolytica* NS18 as evaluated by fluorescence assay (Figs. 2, 4a, b) and GC analysis (Fig. 4b).

### Screening heterologous *DGA1* genes for increased lipid production

Given the effectiveness of endogenous DGA1 overexpression in *Y. lipolytica*, we next sought to express DGA1 from oleaginous organisms that natively attain lipid at 50 % or more of their dry weight and have publically accessible genome sequences [3, 37–40]. We chose *DGA1* genes from five such organisms: *Rhodosporidium toruloides, Lipomyces starkeyi, Aurantiochytrium limacinum, Aspergillus terreus,* and *Claviceps purpurea*. Three versions of the *R. toruloidesDGA1* gene were expressed in *Y. lipolytica*: NG49—the native *R. toruloidesDGA1* gene amplified from *R. toruloides* genomic DNA; NG66—a synthetic gene containing *R. toruloides* DGA1 cDNA without introns; and NG67—a synthetic gene containing *R. toruloides* DGA1 cDNA without introns and codon optimized for expression in *Y. lipolytica.* Two versions of *L. starkeyi* DGA1 were expressed in *Y. lipolytica*: NG68—a synthetic gene containing *L. starkeyi* DGA1 cDNA without introns; and NG69—a synthetic gene containing *L. starkeyi* DGA1 cDNA without introns and codon optimized for expression in *Y. lipolytica. A. limacinum* (NG70)*, A. terreus* (NG71), and *C. purpurea* (NG72) *DGA1* genes were synthesized without introns and codon optimized for expression in *Y. lipolytica*. A total of nine different DGA1 genes were expressed in *Y. lipolytica* under the same strong *Y. lipolytica* GPD1 promoter (Table 2). Expression of all nine *DGA1* genes had a positive effect on lipid content in *Y. lipolytica* (Fig. 2). Due to the random, non-targeted nature of integration, which can result in a range of DGA1 expression, the diversity we saw in transformants’ lipid accumulation was not unexpected. Additionally, there is the potential for multiple DGA1 expression vector copies to simultaneously integrate during genetic transformation. To investigate the likelihood of our results being influenced by DNA copy number, we performed an experiment where two independent, non-linked selectable markers were co-transformed and the frequency of selectable marker co-expression was measured (Additional file 1: Figure S1). This test indicated that for the DNA loadings used in this study (approximately 1 μg vector DNA/transformation) the frequency of two copies simultaneously integrating is 6 % of total transformants.

Transformants that demonstrated the highest fluorescence output per optical density were generated by overexpression of *R. toruloides* DGA1 (NG66, NG67) and *L. starkeyi* DGA1 (NG68). These achieved lipid levels approximately three-fold higher than NS18 and were also higher than transformants overexpressing the native *Y. lipolyticaDGA1* gene. The most effective *DGA1* genes came from donors that were repeatedly reported to attain the highest lipid contents among other oleaginous yeast strains [3, 41]. These results suggest that DGA1-specific activity and/or expression level could be a major factor driving lipid production levels in oleaginous yeasts. The effect of native *R. toruloides* DGA1 (NG49) overexpression on lipid production in *Y. lipolytica* was not as high as the effect of the synthetic versions of *R. toruloidesDGA1* genes that did not contain introns. This result may indicate that the heterologous *R. toruloidesDGA1* gene was not spliced efficiently in *Y. lipolytica*. Codon optimization of *R. toruloides* and *L. starkeyiDGA1* genes did not have a positive effect on lipid production over the native cDNA sequences.

The top lipid-producing isolate expressing the *R. toruloidesDGA1* gene was designated NS281. Strains expressing DGA1 from *A. limacinum, A. terreus*, and *C. purpurea* did not show substantial improvements in lipid content compared to strains overexpressing the native *Y. lipolyticaDGA1* gene (Fig. 2).

### Screening additional genetic targets for increased lipid production in a DGA1 overexpression background

A secondary screen was designed to test the combination of DGA1 overexpression with other targets in the lipid accumulation pathway (Fig. 1). Secondary deletion or overexpression targets, under the control of the *Y. lipolytica* EXP1 promoter, were integrated into strain NS125, which overexpresses the *Y. lipolyticaDGA1* gene under the control of the *Y. lipolytica* TEF1 promoter. Targeted gene deletions were enabled by treatment of cells with hydroxyurea to arrest cell cycle division in the S phase during preparation of competent cells [42]. Table 1 summarizes the relative effect of secondary deletion and overexpression targets on lipid content compared to the parental strain NS125. Four overexpression targets, GPD1, SLC1, DGA2, and LRO1, had a positive effect on lipid content when combined with DGA1. Interestingly, all four of these enzymes are involved in the last steps glycerol backbone synthesis and fatty acid attachment for triacylglycerol synthesis. Overexpression of the *Y. lipolyticaDGA2* gene increased lipid content by nearly 65 % compared to the parental strain NS125, which was the highest increase among the targets tested. Due to a limited number of dominant genetic markers, DGA2 alone was selected for further investigation. DGA2 is located in the endoplasmic reticulum and is hypothesized to be responsible for TAG formation in newly formed lipid bodies, while DGA1 is located on the lipid body membrane and its hypothesized function is to accumulate TAG within the same lipid body and increase its volume [43]. Considering that overexpression of homologous DGA2 alone in *Y. lipolytica* did not have as pronounced of an effect on lipid content (data not shown), the above data suggest that native DGA2 function is not limiting for TAG formation in NS18 wild-type *Y. lipolytica* but may become a limiting factor once DGA1 activity is abundant.

**Table 1:** Relative lipid contents of strains with overexpressed or deleted genetic targets in addition to DGA1 overexpression

Genetic background		Function	(Fl/OD)_mutant_/(Fl/OD)_NS125_
WT		Wild-type *Yarrowia lipolytica* strain	0.55 ± 0.02
DGA1 (NS125)		Diacylglycerol acyltransferase; catalyzes the terminal step of triacylglycerol (TAG) formation, acylates diacylglycerol using acyl-CoA as an acyl donor	1.00 ± 0.09
DGA1	Δgut2	Mitochondrial glycerol-3-phosphate dehydrogenase	0.95 ± 0.12
DGA1	GPD1	NAD-dependent glycerol-3-phosphate dehydrogenase	1.12 ± 0.03
DGA1	ME	Mitochondrial malic enzyme; catalyzes the oxidative decarboxylation of malate to pyruvate	0.92 ± 0.15
DGA1	ACL1/2	ATP citrate lyase 1 & 2	0.99 ± 0.09
DGA1	ACC1	Acetyl-CoA carboxylase subunit 1	1.05 ± 0.19
DGA1	SCT1	Bifunctional glycerol-3-phosphate/glycerone-phosphate O-acyltransferase	1.03 ± 0.01
DGA1	SLC1	1-acylglycerol-3-phosphate O-acyltransferase	1.25 ± 0.07
DGA1	SCD1	Stearoyl-CoA desaturase. Endoplasmic reticulum (ER) protein that catalyzes the Δ9-cis desaturation of saturated fatty acids	0.85 ± 0.18
DGA1	DGA2	DGAT1 acyl-CoA:diacylglycerol acyltransferase family	1.62 ± 0.03
DGA1	LRO1	Acyltransferase that catalyzes diacylglycerol esterification; one of several acyltransferases that contribute to triglyceride synthesis	1.33 ± 0.07
DGA1	Δtgl3	Bifunctional triacylglycerol lipase. Major lipid particle-localized triacylglycerol (TAG) lipase	0.79 ± 0.24
DGA1	Δtgl4	Multifunctional lipase/hydrolase/phospholipase; triacylglycerol lipase, steryl ester hydrolase, and Ca2 + -independent phospholipase A2	1.03 ± 0.08
DGA1	Δmfe1	Multifunctional enzyme, member of the peroxisomal hydroxyacyl coenzyme A dehydrogenase family	0.89 ± 0.03

Lipid content was measured by fluorescence-based lipid assay after 96 h of fermentation in 48-well plates and normalized by the value corresponding to the parental strain NS125 overexpressing DGA1 alone. All genes described here were amplified from *Y. lipolytica* genomic DNA and sequences are given in Additional file 1

### Screening heterologous DGA2s for increased lipid production in a DGA1 overexpression background

In order to identify a DGA2 with higher activity and/or expression than that of *Y. lipolytica*, *DGA2* genes (Table 2) from six donors (*Y. lipolytica*, *R. toruloides*, *L. starkeyi*, *A. terreus*, *C. purpurea*, *Chaetomium globosum*) were expressed in *Y. lipolytica* strains NS125 and NS281 (overexpressing *Y. lipolytica* and *R. toruloidesDGA1* genes, respectively).

**Table 2:** Description of DGA1 and DGA2 genes overexpressed in *Yarrowia lipolytica*

Gene	Source	Description
NG15	*Yarrowia lipolytica*	native *Y. lipolytica* DGA1 gene amplified form *Y. lipolytica* genomic DNA
NG49	*Rhodosporidium toruloides*	native *R. toruloides* DGA1 gene amplified form *R. toruloides* genomic DNA
NG66	*Rhodosporidium toruloides*	synthetic gene containing *R. toruloides* DGA1 cDNA without introns
NG67	*Rhodosporidium toruloides*	synthetic gene containing *R. toruloides* DGA1 cDNA without introns and codon optimized for expression in *Y. lipolytica*
NG68	*Lipomyces starkeyi*	synthetic gene containing *L. starkeyi* DGA1 cDNA without introns
NG69	*Lipomyces starkeyi*	synthetic gene containing L. starkeyi DGA1 cDNA without introns and codon optimized for expression in *Y. lipolytica*
NG70	*Aurantiochytrium limacinum*	synthetic gene containing *A. limacinum* DGA1 cDNA without introns and codon optimized for expression in *Y. lipolytica*
NG71	*Aspergillus terreus*	synthetic gene containing *A. terreus* DGA1 cDNA without introns and codon optimized for expression in *Y. lipolytica*
NG72	*Claviceps purpurea*	synthetic gene containing *C. purpurea* DGA1 cDNA without introns and codon optimized for expression in *Y. lipolytica*
NG16	*Yarrowia lipolytica*	native *Y. lipolytica* DGA2 gene amplified form *Y. lipolytica* genomic DNA
NG109	*Rhodosporidium toruloides*	synthetic gene containing *R. toruloides* DGA2
NG110	*Lipomyces starkeyi*	synthetic gene containing *L. starkeyi* DGA2
NG111	*Aspergillus terreus*	synthetic gene containing *A. terreus* DGA2
NG112	*Claviceps purpurea*	synthetic gene containing *C. purpurea* DGA2
NG113	*Chaetomium globosum*	synthetic gene containing *C. globosum* DGA2

Gene sequences are given in Additional file 1

**Fig. 3:**
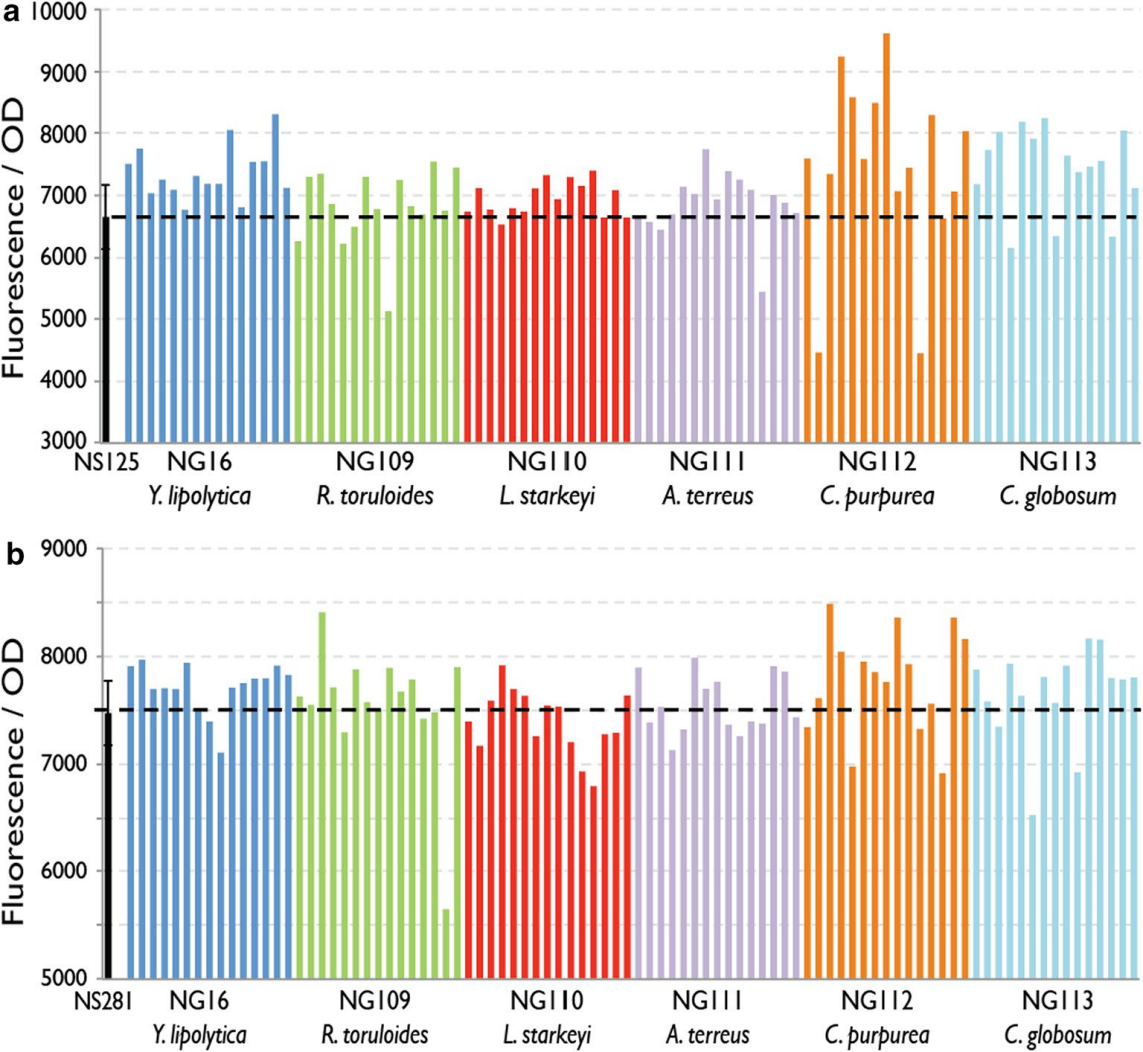
Overexpression of *DGA2* genes in *Y. lipolytica*. Six *DGA2* genes (Table 2) under the control of the *Y. lipolytica* TEF1 promoter were overexpressed in NS125 (**a**) and NS281 (**b**) strains that overexpress DGA1 from *Y. lipolytica* and *R. toruloides*, respectively. Fifteen transformants for each gene were analyzed by fluorescence-based lipid assay after 72 h of growth in nitrogen-limited media. The average with standard deviation from triplicate experiments is shown for the parent strain. Fluorescence was measured at excitation 486 nm and emission 510 nm and normalized by cell optical density (OD) at 600 nm

The effect of different *DGA2* genes on lipid content measured by fluorescence-based lipid assay is shown in Fig. 3. The effect of DGA2 overexpression was more noticeable in the NS125 strain background (Fig. 3a), possibly due to lower baseline lipid content in this strain than in NS281 (Fig. 3b). In both NS125 and NS281 strains, *C. purpurea* DGA2 (NG112) overexpression yielded the highest increase in lipid content.

### Deletion of TGL3 increased lipid accumulation at the end of fermentation

**Fig. 4:**
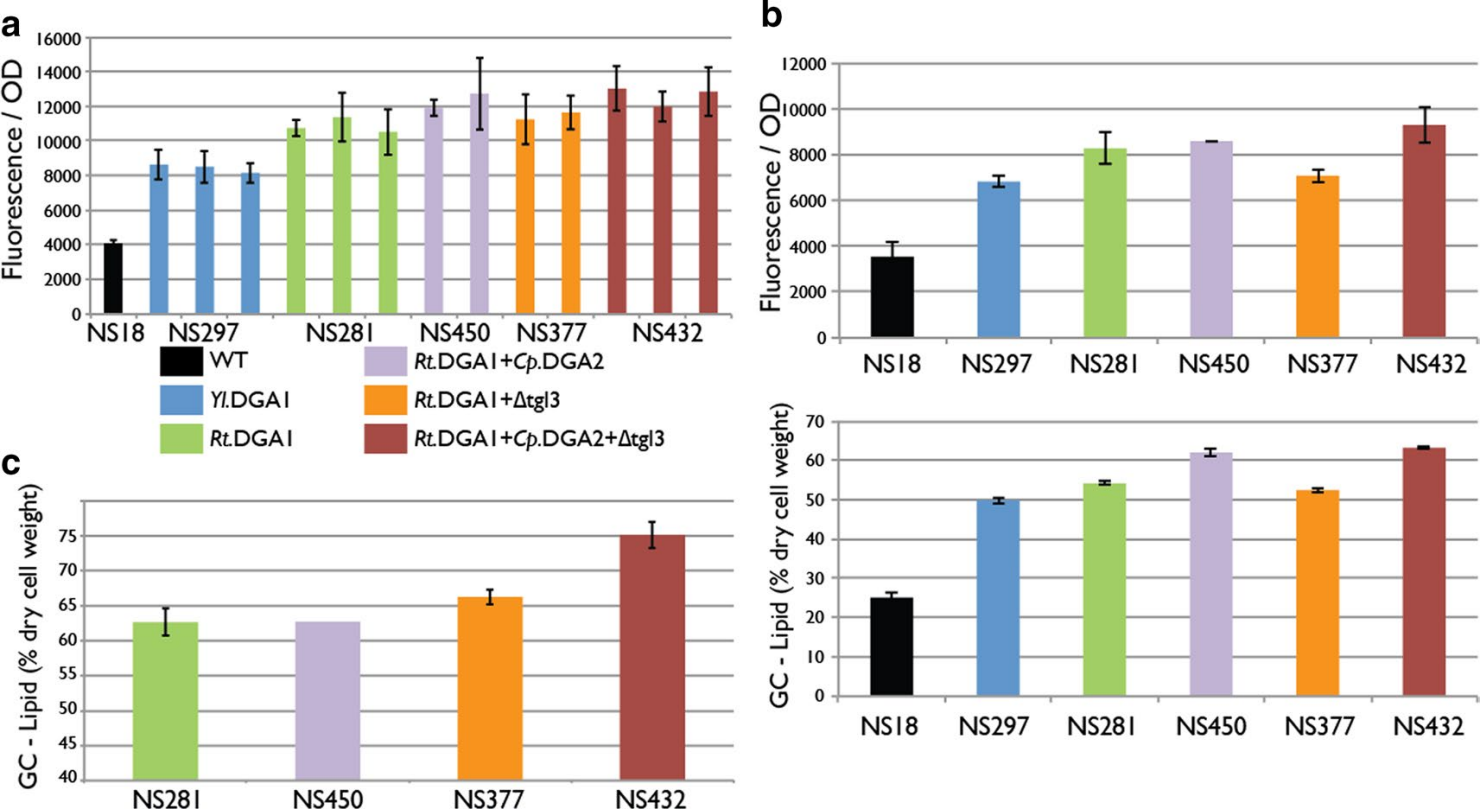
Comparison of lipid accumulation in *Y. lipolytica* strains with different target combinations by different methods. **a** Strains analyzed by fluorescence assay after 96 h of fermentation in a 48-well plate. Two or three transformants were analyzed for each construct and average with standard deviation is shown. **b** Strains analyzed by fluorescence assay and GC after 96 h of fermentation in 50-mL flasks. The measurement was done in triplicates and average with standard deviation is shown. **c** Strains analyzed by GC after 140 h of fermentation in 1 L bioreactors. With exception for NS450 the measurement was done in duplicates and average with standard deviation is shown

TGL3 is one of two intracellular lipases responsible for the first step of lipid body localized TAG degradation in *Y. lipolytica* [16, 31]. It has been suggested that TGL3 positively regulates TGL4, the primary lipase, but may not have lipase activity on its own, as it lacks a key amino acid motif that is specific for lipase activity [31]. TGL3 or TGL4 deletions in the NS125 background did not increase lipid content after 96 h of fermentation in smaller scale experiments (Table 1). However, as these genes are directly or indirectly involved in the breakdown of TAG, we hypothesized that their activity would be critical at the end of the bioprocess, when carbon is exhausted and cells attempt to recover stored carbon from lipids. We compared lipid levels at late time-points in the strain overexpressing *R. toruloides* NG66 DGA1 alone (NS281) and the strain combining NG66 overexpression and TGL3 deletion (NS377). Strain NS377 accumulated higher lipid content than NS281 after 140 h of fermentation both in 50-mL shake flasks (data not shown) and in 1 L bioreactors (Fig. 4c). These results indicate that down-regulation of the TAG degradation pathway is beneficial for lipid content late in the bioprocess. Strains with a TGL4 deletion also had a positive effect on lipid content late in the fermentation but reduced overall lipid titer due to a negative effect on overall cell growth (data not shown).

### Combination of the best lipid accumulation genetic modifications

In order to combine a TGL3 deletion with the *R. toruloides* DGA1 and *C. purpurea DGA2* gene overexpressions, *C. purpurea* DGA2 was overexpressed in strain NS377 resulting in strain NS432 (genotype: *Rt*DGA1 *natCp*DGA2 *zeo* tgl3Δ::*hyg*). NS432 reached higher lipid contents than any of the previously generated strains (Fig. 4). As expected from our results with NS377, when grown in 48-well plates or 50-mL flasks for 96 h, NS432 did not demonstrate lipid-content increase over NS450, a strain carrying only the overexpression targets *R. toruloides* DGA1 and *C. purpurea* DGA2 (Fig. 4a, b). Consistent with a late fermentation role for TGL3 in TAG degradation, NS432 achieved a significantly higher lipid content compared to NS450 when grown for 140 h in 50-mL shake flasks (data not shown) or 1-L bioreactors (Fig. 4c).

**Fig. 5:**
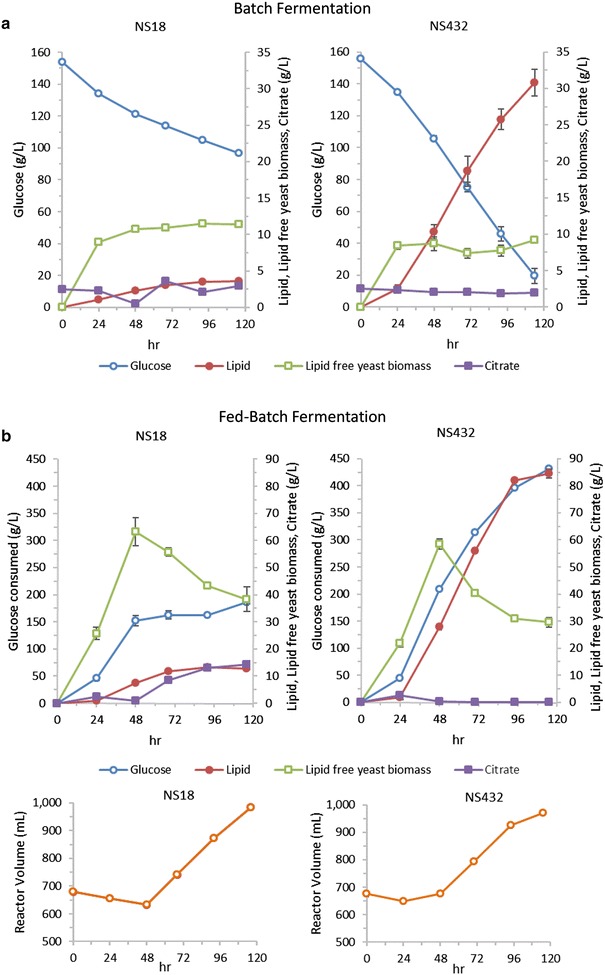
Evaluation of high lipid *Y. lipolytica* strain NS432 performance in 1 L bioreactor as compared to the parental strain NS18 in batch (**a**) and fed-batch (**b**) fermentation. Dry cell weight, glucose consumption, citrate and lipids (reported as fatty acid methyl esters) were monitored throughout the fermentation. All experiments except for NS18—batch were performed twice and the average with standard deviation from duplicate experiments are shown

To study in depth the productivity and the yield that could be reached with NS432, two sets of conditions were explored in 1-L bioreactors. Figure 5 shows batch (a) and fed-batch (b) glucose fermentations of NS432 and wild-type NS18. The calculated lipid production parameters are shown in Table 3. Data in Fig. 5 and Table 3 demonstrate that there is a trade-off between biomass and lipid production for the two different processes. In the batch bioprocess (carbon to nitrogen ratio of approximately 180), NS432 reached a lipid content of 77 % and a yield 0.21 g lipid per g glucose. In the fed-batch fermentation with a carbon to nitrogen ratio of approximately 110, NS432 produced a higher lipid free cell biomass resulting in an 85 g/L lipid titer and 0.73 g/L/h productivity, although at a slightly lower lipid content (73 %) and yield (0.20 g/g) than in the batch process.

**Table 3:** Lipid production parameters calculated for the final time point (120 h) of the batch and fed-batch fermentation experiments with strains NS18 and NS432

	NS18		NS432	
	Batch	Fed-batch	Batch	Fed-batch
Hours post-inoculation	116	116	114	115
Lipid content (% w lipid/w DCW)	24 %	25 % ± 4 %	77 % ± 2 %	73 % ± 2 %
Titer (g lipid/L)	3.6	12.8 ± 1.0	30.8 ± 1.8	84.5 ± 1.7
Productivity (g lipid L^−1^ h^−1^)	0.03	0.11 ± 0.01	0.27 ± 0.02	0.73 ± 0.01
Cell specific productivity^a^ (g lipid g lipid free DCW^−1^ h^−1^)	0.002	0.003 ± 0.001	0.048 ± 0.006	0.044 ± 0.001
Yield (g lipid/g glucose consumed)^b^	0.06	0.07 ± 0.01	0.21 ± 0.004	0.20 ± 0.004

All experiments except for NS18—batch were performed twice and the average with standard deviation from duplicate experiments is shown

^a^ Cell specific productivity calculated over 20–70-h post-inoculation

^b^ Yields calculated for batch cultivation account for a measured 10 % volume loss due to sampling and aeration over the course of fermentation

The stoichiometric pathway yield for TAG lipid production in *Y. lipolytica* based on known biochemistry for this organism has been calculated at 0.276 g per gram glucose [44]. However, this value does not include the expenditure of glucose to produce and maintain non-storage lipid yeast biomass, an important consideration for applied microbial lipid production since the amount of non-lipid biomass (i.e., catalyst) and its cell specific lipid productivity determines the rate of lipid production. As shown in Table 4, this study adds to recent literature indicating that *Y. lipolytica* can be engineered for highly effective lipid production. The batch lipid yield, fed-batch titer, and productivity reported here are among the highest reported [22, 23, 44] and approaches the maximal values predicted by *Y. lipolytica* lipid biochemistry and co-production of non-storage lipid biomass.

**Table 4:** Recently reported literature values for lipid titer, yield, and productivity with engineered *Yarrowia lipolytica* strains

Parental strain	Strain	Lipid pathway genetic engineering	Process	Sugar consumed (g/L)	Lipid titer (g/L)	Overall yield (g/g)	Overall volumetric productivity (g/L/h)	Cell specific productivity (g/g/h)	Reference
po1g	MTYL037	control strain (ura + lacZ)	Batch	80	2.5	0.03	0.02	0.002	[19]
po1g	MTYL065	DGA1 ACC1	Batch	80	17.6	0.20	0.14	0.017	[19]
po1g	YL-ad9	DGA1 ACC1 SGD1	Fed-batch	235	55	0.23	0.71	0.038	[22]
po1f	po1f leu + ura +	control strain	Batch	80	3	0.04	0.02	0.001	[21]
po1f	pex10 mfe1 leu + ura + DGA1	DGA1 Δpex10 Δmfe1	Batch	80	16.1	0.20	0.11	0.017	[21]
po1f	pex10 mfe1 leu + ura + DGA1	DGA1 Δpex10 Δmfe1	Batch	160	25.3	0.16	0.21	–	[21]
po1f	L36 DGA1 leu + ura +	DGA1 mga2-G643R	Fed-batch	160	25	0.21	0.13	0.018	[24]
po1f	E26E1	DGA1 Δpex10 Δmfe1 uga-P209S	Batch	160	39.1	0.24	0.56	0.035	[23]
po1d	JMY2593	control strain (SUC2)	Batch	60	2.6	0.04	0.04	0.004	[26]
po1d	JMY3582	DGA2(x3) Δdga1 Δdga2 Δlro1 Δare1	Batch	60	6.5	0.11	0.09	0.010	[26]
NS18	NS18	wild-type	Batch	60	3.6	0.06	0.04	0.002	this study
NS18	NS432	DGA1 DGA2 Δtgl3	Batch	140	30.8	0.21	0.27	0.048	this study
NS18	NS432	DGA1 DGA2 Δtgl3	Fed-batch	430	84.5	0.20	0.73	0.044	this study

Where necessary, values for overall and cell specific productivities were estimated from figures of cited references [24, 26]

## Conclusions

In this study, we describe a strain engineering strategy that applied screening of endogenous and heterologous targets and target combinations for the construction of a high lipid-producing yeast biocatalyst. First, we confirmed that the overexpression of DGA1 increased lipid content in *Y. lipolytica*. A secondary screen identified genes that boosted lipid accumulation when overexpressed or deleted in combination with DGA1 overexpression. We then searched for heterologous *DGA1* and *DGA2* genes that would outperform the native enzymes in lipid accumulation and identified *R. toruloides* DGA1 and *C. purpurea* DGA2. Finally, we found that deletion of TGL3 reduces TAG degradation during the late phase of the bioprocess when nutrients are limited and lipase-mediated TAG mobilization is triggered. The yields, productivities, and titers reported in this study may further support the applied goal of developing *Y. lipolytica* for cost effective microbial lipid production. Additionally, several studies have demonstrated the ability to expand *Y. lipolytica*’s substrate range to low-cost feedstocks such as sucrose [45], xylose [46], cellobiose and lignocellulosic biomass [47], and starch [48]. Researchers have also investigated continuous culture [49, 50] as a lower cost processing method than batch or fed-batch fermentation. The combined actions of attaining high lipid titer with low-cost feedstock utilization and advanced processing strategies should help enable use of this yeast for production of biofuels and biochemicals.

## Methods

### Strains, media, and cultivation methods

Wild-type *Y. lipolytica* strain YB-392 was obtained from the ARS (NRRL) culture collection. For routine maintenance and genetic transformation, strains were cultured in YPD (10 g/L yeast extract, 20 g/L bacto peptone, 20 g/L glucose) with the addition of 20 g/L agar for solid media and 300 μg/mL hygromycin B (Corning), 500 μg/mL nourseothricin (Werner Bioreagents) or 1 mg/mL zeocin (Invitrogen) for antibiotic selection. All *Yarrowia* strains were cultured at 30 °C.

Media and growth conditions used in this study for *Escherichia coli* were previously described by Sambrook and Russell [51], those for *Saccharomyces cerevisiae* by Shanks et al. [52], and those for *Y. lipolytica* by Barth and Gaillardin [9].

Medium used for fluorescence assay in 96-well or 48-well plates, and shake flasks was: urea (0.5 g/L), yeast extract (1.5 g/L), casamino acids (0.85 g/L), YNB without amino acids and ammonium sulfate (1.7 g/L), glucose (100 g/L), potassium hydrogen phthalate buffer adjusted to pH 5.5 (5.1 g/L).

Initial concentrations of medium for 1 L batch and fed-batch bioreactors was: glucose T_0_ (150 g/L), sensient amberex 1003 yeast extract (3 g/L), sensient amberferm 4500 corn peptone (0.1 g/L), KH_2_PO_4_ (4 g/L), MgSO_4_·7H_2_O (2 g/L), CaCl_2_·6H_2_O (0.8 g/L), NaCl (0.4 g/L), thiamine (12 mg/L), biotin (1 mg/L), trace elements [Na_2_MoO_4_·2H_2_O (160 mg/L), CuSO_4_·5H_2_O (0.2 mg/L), H_3_BO_3_ (40 mg/L), MnSO_4_·H_2_O (180 mg/L), FeCl_2_·6H_2_O (75 mg/L), ZnSO_4_·7H_2_O (20 mg/L)], and Antifoam 204 (Sigma-Aldrich) (1 mL/L). For the batch process, (NH_4_)_2_SO_4_ was added at 0.5 g/L and in the fed-batch process at 11 g/L for the initial reactor volume. Process parameters included an inoculum volume of 2 % from an overnight shake flask in YPD, pH control at 3.5 automatically adjusted with 10 N sodium hydroxide, a temperature of 30 °C, aeration at 0.3 vvm air, and agitation at 1000 rpm for both processes. For the fed-batch process a glucose substrate feed was initiated after batch glucose consumption at a rate of 6.5 mL/h from 420 mL of a 75 % w/v concentrated glucose stock solution. The initial batch volume was 680 mL.

### Genetic transformation

*Y. lipolytica* competent cells were prepared following the protocol of Chen et al. [53]. Cells were grown overnight spread on YPD plates, and grown cells were transferred from the plate with 1 mL water. 50 µl was aliquoted per transformation reaction, cells were centrifuged and the supernatant was discarded. 9 µl of each PCR product (without purification) and 92 µl of transformation mix (80 μL 60 % polyethylene glycol 4000, 5 µl 2 M dithiothreitol, 5 µl 2 M lithium acetate pH 6, 2 µl 10 mg/mL single-stranded salmon sperm DNA) was added to the cell pellet. The transformation reaction was mixed well by vortexing and heat shocked at 39 °C for 1 h. Cells were centrifuged, the supernatant discarded, cells were resuspended in 1 mL YPD, transferred to culture tubes and cultured overnight before plating on selective media (YPD containing hygromycin B, zeocin or nourseothricin).

### Plasmid construction

#### Gene overexpression

Standard molecular biology techniques were used in this study [51]. Restriction enzymes and DNA polymerase were purchased from New England Biolabs (Ipswich, MA). Descriptions of plasmids used for gene overexpressions and gene sequences are presented in Additional file 1.

For DGA1 overexpressions, the linear expression construct included an expression cassette for different *DGA1* genes and for the *nat1* gene used as a marker for selection of random chromosomal integrants with nourseothricin. For DGA2 overexpressions, the linear expression construct included an expression cassette for *DGA2* genes and for the *ble* gene used as a marker for selection with zeocin. For each expression construct 15 transformants were analyzed by fluorescence lipid assay.

For overexpression of secondary target genes, the linear expression construct included an expression cassette for the gene of interest and for the *hph* gene used as a marker for selection with hygromycin.

### Gene deletion in *Yarrowia lipolytica*

The *Y. lipolyticaTGL3*, *GUT2*, *TGL4* and *MFE1* genes were deleted as follows: A two-fragment deletion cassette was amplified by PCR from a plasmid containing the hygromycin resistance gene *hph* such that the *hph* gene was split into two fragments that overlapped and were flanked by ~50 bp of homology to the upstream and downstream regions of the coding sequence (Primers summarized on Additional file 1: Table S1). The resulting PCR fragments were co-transformed. *Y. lipolytica* grown overnight on solid YPD media was used to inoculate a 25 mL YPD culture at OD_600_ = 0.5. After 3 h of growth, 50 mM hydroxyurea was added [42]. Cells were harvested 2 h post hydroxyurea addition, and the standard transformation protocol was followed with washed cell pellets. Hygromycin resistant colonies were screened by PCR to confirm the absence of the gene and the presence of a specific product.

### Lipid content measurement by fluorescence assay

Strains patched on YPD agar media were inoculated into fluorescent assay media volumes of 1.5 mL for 24-well plates or 0.6 mL for 96-well plates or 50 mL for shake flasks. Plates were incubated at 30 °C for 96 h and 900 rpm, at 70–90 % humidity in an Infors Multitron ATR shaker. The shake flasks were incubated at 30 °C for 96 h and 200 rpm in a New Brunswick Scientific shaker. To measure fluorescence, 20 μL cells were mixed with 20 μL of 100 % ethanol in microplates and incubated at 4 °C for 30 min. A master mix containing 1 M potassium iodide, 1 mM bodipy 493/503, 0.5 µL dimethyl sulfoxide, 1.5 µL of 60 % PEG 4000 and 27 µL water was then aliquoted into Costar black well/clear bottom plates (80 μL/well) and 20 μL of the ethanol/cell mix was added. Fluorescence was measured with a SpectraMax M2 spectrophotometer (molecular devices) at excitation 484 nm and emission 510 nm. The optical density (OD) at 600 nm was measured in the same plate. Fluorescence correlated with lipid content measured by GC.

### Lipid extraction and GC analysis

Dried biomass, prepared by lyophilization of 1 mL fermentation samples washed with an equal volume of water, was subjected to acid-catalyzed transesterification with 0.5 N hydrochloric acid in methanol (20 × 1 mL ampule, Sigma) at 85 °C for 90 min. After the transesterification was completed, the lipid-soluble components of the reaction mixture were separated from the water-soluble components using a two-phase liquid extraction by adding water and isooctane and subsequently analyzed with a capillary gas chromatograph (GC) equipped with a robotic injector, flame ionization detector (Thermo Scientific Trace GC Ultra with AS 3000 autosampler) and HP-INNOWAX capillary column (30 m × 0.25 mm × 0.15 micrometers, Agilent). Quantification of the methyl-ester products was achieved with use of both an internal standard (13:0 or 15:0 fatty acid) and various concentrations of an external standard mixture (NHI-D, Supelco Analytical, Bellefonte, PA) of fatty acid methyl esters (FAMEs). Lipids were reported as FAME equivalents of 16:0, 16:1, 18:0, 18:1, and 18:2 fatty acids, e.g., the amount of FAME resulting from lipid extraction and transesterification after accounting for transesterification efficiency via the internal standard.

## Additional file

10.1186/s13068-016-0492-3 Contains a description of the multi-copy integration experiment, gene overexpression vectors, lipid biosynthesis pathway gene sequences used in this study, and primers used to construct gene knockout targets.

## Abbreviations

TAG: triacylglycerol
GC: gas chromatography
cDNA: complementary DNA
YPD: yeast extract, peptone, dextrose medium
YNB: yeast nitrogen base medium
FAME: fatty acid methyl ester
OD: optical density
WT: wild-type organism
