# Supplementary material for: Engineering of a high lipid producing Yarrowia lipolytica strain
Source: Biotechnol Biofuels. 2016 Mar 31;9:77. doi: 10.1186/s13068-016-0492-3 (PMC4815080; doi:10.1186/s13068-016-0492-3)
Supplement: Supplementary file 1 — 10.1186/s13068-016-0492-3 Contains a description of the multi-copy integration experiment, gene overexpression vectors, lipid biosynthesis pathway gene sequences used in this study, and primers used to construct gene knockout targets. [file 13068_2016_492_MOESM1_ESM.docx]

Additional data file 1


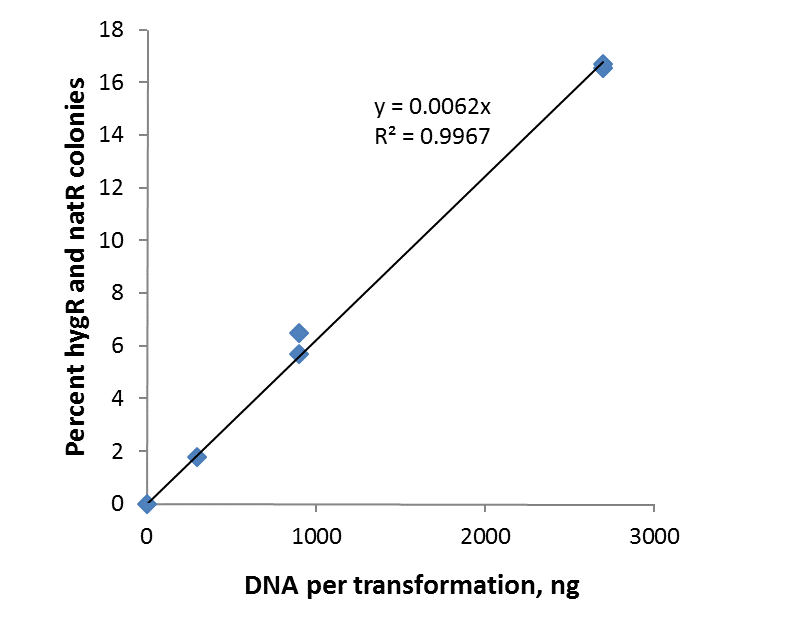


**Additional data file, figure 1. Frequency of two independent, non-linked selectable markers integrating during the same transformation event.** *Y. lipolytica* NS18 was transformed with an equal mixture of linear DNA containing either a hygR or natR selectable marker. Total DNA loading per transformation were 150 ng, 500 ng, and 1350 ng, and colonies were counted by either plate selection of hygromycin and nourseothricin containing agar plates, or by plate selection on hygromycin agar plates and replica plating to nourseothricin containing agar plates.

Map of the DNA vector used to overexpress different DGA1 genes in *Y. lipolytica* strain NS18. The vector was linearized by PacI/NotI restriction digest before transformation.

2u ori – *S. cerevisiae* origin of replication from 2μ circle plasmid

pMB1 ori – *E. coli* pMB1 origin of replication from pBR322 plasmid

AMP_R - *bla* gene used as marker for selection with ampicillin

YlGpd1p *- Y. lipolytica* GPD1 promoter -931 to -1

YlCYC1t - *Y. lipolytica* CYC1 terminator 300 bp after stop

ScTEF1p - *S. cerevisiae* TEF1 promoter -412 to -1

NAT_R - *Streptomyces noursei* *nat1* gene used as marker for selection with nourseothricin

ScCYC1t - *S. cerevisiae* CYC1 terminator 275 bp after stop

Sc URA3 - *S. cerevisiae* URA3 auxotrophic marker for selection in yeast.

Map of the DNA vector used to overexpress different DGA2 genes in *Y. lipolytica* strains NS125 and NS281. The vector was linearized by PacI/AscI restriction digest before transformation.

2u ori – *S. cerevisiae* origin of replication from 2μ circle plasmid

pMB1 ori – *E. coli* pMB1 origin of replication from pBR322 plasmid

AMP_R - *bla* gene used as marker for selection with ampicillin

YlTEF1p1 – *Y. lipolytica* TEF1 promoter -406 to -1

YlCYC1t - *Y. lipolytica* CYC1 terminator 300 bp after stop

YlTEF1p2 - *Y. lipolytica* TEF1 promoter -406 to +125

ZEO_R - *Streptoalloteichus hindustanus ble* gene used as marker for selection with Zeocin

YlTEF1t – *Y. lipolytica* TEF1 terminator 400 bp after stop

Sc URA3 - *S. cerevisiae* URA3 auxotrophic marker for selection in yeast.

**SI Figure 4.** Map of the DNA vector used to overexpress the secondary target genes in *Y. lipolytica* strain NS125. The vector was linearized by AscI/PmeI restriction digest before transformation.

2u ori – *S. cerevisiae* origin of replication from 2μ circle plasmid

pMB1 ori – *E. coli* pMB1 origin of replication from pBR322 plasmid

AMP_R - *bla* gene used as marker for selection with ampicillin

YlEXP1p – *Y. lipolytica* EXP1 promoter -999 to -1

YlCYC1t - *Y. lipolytica* CYC1 terminator 300 bp after stop

ScFBA1p – *S. cerevisiae* FBA1 promoter -822 to -1

HYG_R - *hph* gene used as marker for selection with Hygromycin B

ScFBA1t – S. cerevisiae FBA1 terminator 205 bp after stop

Sc URA3 – *S. cerevisiae* URA3 auxotrophic marker for selection in yeast.

| **Target** | **Position** | **Name** | **Squence** |
| --- | --- | --- | --- |
| **TGL3** | 5’ fragment | NP1798 external Fd | CAGCTCTCTTCCCCCGTTCAGCTCCTTTTCTACCGCGATTATGAAGAAGCCCGAGCTGAC |
|  |  | **NP655 internal Re** | ACCACTTGGCGAGACTTCATCTGT |
|  | 3’ fragment | **NP656 internal Fd** | AGCATCGTCAAAGTTGCCATCCAC |
|  |  | NP1799 external Re | TTTAGTCTCATCGTTAGTAGTTATGTGCTCTGCTCGGGGTTACTCCTTAGCTCGAGGTCG |
| **GUT2** | 5’ fragment | NP1563 external Fd | tcaatgtagacgtcgcggaacTTCTCTTTCCTACCCACCACCCCAAACAAATGAAGAAGCCCGAGCTGACC |
|  |  | **NP655 internal Re** | ACCACTTGGCGAGACTTCATCTGT |
|  | 3’ fragment | **NP656 internal Fd** | AGCATCGTCAAAGTTGCCATCCAC |
|  |  | NP1800 external Re | CAAGTGATTATCTTATTGGCTATACGCTTTTACTATACAGTTACTCCTTAGCTCGAGGTCG |
| **TGL4** | 5’ fragment | NP2203 external Fd | CAACTGTAGAAGCTTTGAGACACAGTCCAACGGGTAGGAATGAAGAAGCCCGAGCTGACC |
|  |  | **NP655 internal Re** | ACCACTTGGCGAGACTTCATCTGT |
|  | 3’ fragment | **NP656 internal Fd** | AGCATCGTCAAAGTTGCCATCCAC |
|  |  | NP2204 external Re | TTTAAAGCTGTCTAATTGCCATGAATGAAGACCGTTGCGGTTACTCCTTAGCTCGAGGTCGAG |
| **MFE1** | 5’ fragment | NP956 external Fd | ctgccgtcaaggcttttggc |
|  |  | **NP655 internal Re** | ACCACTTGGCGAGACTTCATCTGT |
|  | 3’ fragment | **NP656 internal Fd** | AGCATCGTCAAAGTTGCCATCCAC |
|  |  | NP957 external Re | ttggtaggaataggccactgtcgg |

Primers used for gene deletion. Each deletion cassette was amplified as two overlapping fragments of the *hph* gene. The same internal primers homologous to *hph* gene were used for each target. External primers were specific to each target.

Gene sequences used in this study

| Yarrowia lipolytica gene | Gene Identification | |
| --- | --- | --- |
| DGA1 | YALI0E32769g | |
| GUT2 | YALI0B13970g | |
| GPD1 | YALI0B02948g | |
| ME | YALI0E18634g | |
| ACL1/ACL2 | YALI0E34793g | YALI0D24431g |
| ACC1 | YALI0C11407g | |
| SCT1 | YALI0C00209g | |
| SLC1 | YALI0E18964g | |
| SCD1 | YALI0C05951g | |
| DGA2 | YALI0D07986g | |
| LRO1 | YALI0E16797g | |
| TGL3 | YALI0D17534g | |
| TGL4 | YALI0F10010g | |
| MFE1 | YALI0E15378g | |

| Novogy Gene ID | Gene DNA sequence |
| --- | --- |
| NG15 | ATGACTATCGACTCACAATACTACAAGTCGCGAGACAAAAACGACACGGCACCCAAAATCGCGGGAATCCGATATGCCCCGCTATCGACACCATTACTCAACCGATGTGAGACCTTCTCTCTGGTCTGGCACATTTTCAGCATTCCCACTTTCCTCACAATTTTCATGCTATGCTGCGCAATTCCACTGCTCTGGCCATTTGTGATTGCGTATGTAGTGTACGCTGTTAAAGACGACTCCCCGTCCAACGGAGGAGTGGTCAAGCGATACTCGCCTATTTCAAGAAACTTCTTCATCTGGAAGCTCTTTGGCCGCTACTTCCCCATAACTCTGCACAAGACGGTGGATCTGGAGCCCACGCACACATACTACCCTCTGGACGTCCAGGAGTATCACCTGATTGCTGAGAGATACTGGCCGCAGAACAAGTACCTCCGAGCAATCATCaCCACCATCGAGTACTTTCTGCCCGCCTTCATGAAACGGTCTCTTTCTATCAACGAGCAGGAGCAGCCTGCCGAGCGAGATCCTCTCCTGTCTCCCGTTTCTCCCAGCTCTCCGGGTTCTCAACCTGACAAGTGGATTAACCACGACAGCAGATATAGCCGTGGAGAATCATCTGGCTCCAACGGCCACGCCTCGGGCTCCGAACTTAACGGCAACGGCAACAATGGCACCACTAACCGACGACCTTTGTCGTCCGCCTCTGCTGGCTCCACTGCATCTGATTCCACGCTTCTTAACGGGTCCCTCAACTCCTACGCCAACCAGATCATTGGCGAAAACGACCCACAGCTGTCGCCCACAAAACTCAAGCCCACTGGCAGAAAATACATCTTCGGCTACCACCCCCACGGCATTATCGGCATGGGAGCCTTTGGTGGAATTGCCACCGAGGGAGCTGGATGGTCCAAGCTCTTTCCGGGCATCCCTGTTTCTCTTATGACTCTCACCAACAACTTCCGAGTGCCTCTCTACAGAGAGTACCTCATGAGTCTGGGAGTCGCTTCTGTCTCCAAGAAGTCCTGCAAGGCCCTCCTCAAGCGAAACCAGTCTATCTGCATTGTCGTTGGTGGAGCACAGGAAAGTCTTCTGGCCAGACCCGGTGTCATGGACCTGGTGCTACTCAAGCGAAAGGGTTTTGTTCGACTTGGTATGGAGGTCGGAAATGTCGCCCTTGTTCCCATCATGGCCTTTGGTGAGAACGACCTCTATGACCAGGTTAGCAACGACAAGTCGTCCAAGCTGTACCGATTCCAGCAGTTTGTCAAGAACTTCCTTGGATTCACCCTTCCTTTGATGCATGCCCGAGGCGTCTTCAACTACGATGTCGGTCTTGTCCCCTACAGGCGACCCGTCAACATTGTGGTTGGTTCCCCCATTGACTTGCCTTATCTCCCACACCCCACCGACGAAGAAGTGTCCGAATACCACGACCGATACATCGCCGAGCTGCAGCGAATCTACAACGAGCACAAGGATGAATATTTCATCGATTGGACCGAGGAGGGCAAAGGAGCCCCAGAGTTCCGAATGATTGAGTAA |
| NG49 | atgggccagcaggcgacgcccgaggagctatacacacgctcagagatctccaagatcaagcaagtcgagccagctcttctcctcaccaccccacaacataccccgcagcccacgacagccctcccacagcacctgcagcctgctgaccagctcgagaacacccacagattcgcaccctttggcgtcccgcggtcgcgccggctgcagaccttctccgtctttgcctggacgacggcactgcccatcctactcggcgtcttcttcctcctctggtgcgtcaggcttggcgtgatctgagagtagcgggcggatcatctgacctgcttcttcgctgcagctcgttcccaccgctctggccggctgtcattgcctacctcacctgggtctttttcattgaccaggcgccgattcacggtggacgggcgcagtcttggctgcggaagagtcggatatgggtctggtttgcaggatactatcccgtcaggtgcgtcctctttccaagcctgcgtctcgaggcctcgctcacggccaactcgcccgaccggctacctccgaactttccgtcaacagcttgatcaaggtcagtctgcgcgtctctcgacttcagtgctctgtggaggagctgcgccattgggcccgacctgcggagggcctcaaaggacgatgccgctgacttcctttcctccgacagagcgccgacttgccgcctgaccggaagtacgtctttggctaccacccgcacggcgtcataggcatgggcgccatcgccaacttcgcgaccgacgcaaccggcttctcgacactcttccccggcttgaaccctcacctcctcaccctccaaagcaacttcaagctcccgctctaccgcgagttgctgctcgctctcggcatatgctccgtctcgatgaagagctgtcagaacattctgcgacaaggtgagcggtatgcgcaagacgggcggtcaagcgtgaacgcagtgaacgagaagagctgaccttccgccttactccatccgtgcaggtcctggctcggctctcactatcgtcgtcggtggcgccgccgagagcttgagtgcgcatcccggaaccgccgatcttacgctcaagcgacgaaaaggcttcatcaaactcgcgatccggcaaggcgccgaccttgtgcccgtcttttcgttcggcgagaacgacgtgcgcacgctctccgagtctctaaaccggaagcgaatgctgaccgctgcccaattctctctccagatctttggccagctgcgaaacgagcgaggaacgcggctgtacaagttgcagaagcgtttccaaggcgtgtttggcttcaccctccgtacgtctcaccgcgccgtcttgccgaactgctcgttcagtcgctcacgcagctttcactcgcgcagctctcttctacggccggggactcttcaactgtgcgctcgagttcaccgcttcgccaacagcgaggaatgcctccgagtacagcccagctgacgccccatctcttctcatagacaacgtcggattgatgccgtatcgccatccgatcgtctctgtcggtgtgaacccgctctgtcgctcctacctgcgttccttaggctgacaccactcgcgtcaaacagtcggtcgaccaatctcggtagagcagaaggaccacccgaccacggcggacctcgaagaagttcaggcgcggtatatcgcagaactcaagcggtacgttccaagtcgtctgcctccgcttgccgcctcaaataagctgaggcgtgctgaccgtatctgccgaaccgtacagcatctgggaagaatacaaggacgcctacgccaaaagtcgcacgcgggagctcaatattatcgcctga |
| NG66 | atgggccagcaggcgacgcccgaggagctatacacacgctcagagatctccaagatcaaattcgcaccctttggcgtcccgcggtcgcgccggctgcagaccttctccgtctttgcctggacgacggcactgcccatcctactcggcgtcttcttcctcctctgctcgttcccaccgctctggccggctgtcattgcctacctcacctgggtctttttcattgaccaggcgccgattcacggtggacgggcgcagtcttggctgcggaagagtcggatatgggtctggtttgcaggatactatcccgtcagcttgatcaagagcgccgacttgccgcctgaccggaagtacgtctttggctaccacccgcacggcgtcataggcatgggcgccatcgccaacttcgcgaccgacgcaaccggcttctcgacactcttccccggcttgaaccctcacctcctcaccctccaaagcaacttcaagctcccgctctaccgcgagttgctgctcgctctcggcatatgctccgtctcgatgaagagctgtcagaacattctgcgacaaggtcctggctcggctctcactatcgtcgtcggtggcgccgccgagagcttgagtgcgcatcccggaaccgccgatcttacgctcaagcgacgaaaaggcttcatcaaactcgcgatccggcaaggcgccgaccttgtgcccgtcttttcgttcggcgagaacgacatctttggccagctgcgaaacgagcgaggaacgcggctgtacaagttgcagaagcgtttccaaggcgtgtttggcttcaccctccctctcttctacggccggggactcttcaactacaacgtcggattgatgccgtatcgccatccgatcgtctctgtcgtcggtcgaccaatctcggtagagcagaaggaccacccgaccacggcggacctcgaagaagttcaggcgcggtatatcgcagaactcaagcgcatctgggaagaatacaaggacgcctacgccaaaagtcgcacgcgggagctcaatattatcgcctga |
| NG67 | ATGGGACAGCAGGCTACCCCCGAGGAGCTCTACACCCGATCCGAGATTTCTAAGATTAAGTTCGCCCCTTTTGGAGTGCCCCGATCCCGACGACTCCAGACCTTCTCCGTTTTTGCCTGGACCACTGCTCTGCCCATTCTGCTCGGCGTCTTCTTTCTGCTCTGCTCTTTCCCCCCTCTCTGGCCCGCCGTCATCGCTTACCTGACCTGGGTGTTCTTTATCGACCAGGCCCCTATTCACGGCGGTCGAGCTCAGTCCTGGCTGCGAAAGTCTCGAATTTGGGTTTGGTTCGCCGGTTACTACCCCGTCTCTCTCATCAAGTCGGCTGACCTGCCCCCTGATCGAAAGTACGTGTTCGGCTACCACCCTCATGGTGTTATCGGTATGGGAGCCATTGCTAACTTTGCCACCGATGCTACTGGTTTCTCCACCCTCTTTCCCGGACTGAACCCTCACCTGCTCACTCTCCAGTCTAACTTCAAGCTCCCCCTGTACCGAGAGCTGCTCCTGGCCCTGGGTATCTGCTCCGTCTCTATGAAGTCTTGTCAGAACATTCTCCGACAGGGACCTGGTTCGGCTCTGACCATCGTCGTGGGAGGAGCTGCTGAGTCGCTCTCCGCCCATCCTGGAACCGCTGACCTCACTCTGAAGCGACGAAAGGGCTTCATCAAGCTCGCCATTCGACAGGGTGCTGACCTGGTGCCCGTTTTCTCCTTTGGAGAGAACGATATTTTCGGCCAGCTGCGAAACGAGCGAGGAACCCGACTCTACAAGCTGCAGAAGCGATTTCAGGGTGTGTTCGGCTTCACCCTCCCTCTGTTCTACGGACGAGGCCTCTTTAACTACAACGTTGGACTGATGCCCTACCGACACCCTATCGTCTCGGTTGTCGGCCGACCCATTTCCGTGGAGCAGAAGGACCATCCTACCACTGCCGATCTCGAGGAGGTGCAGGCCCGATACATCGCTGAGCTGAAGCGAATTTGGGAGGAGTACAAGGACGCCTACGCTAAGTCTCGAACCCGAGAGCTGAACATCATTGCCTAA |
| NG68 | ATGAGTGAGAAGGCAGAGATCGAGGTTCCGCCGCAAAAATCGACATTCCCTCGCAGTGTGCACTTCGCTCCACTTCATATTCCACTGGAGAGACGCCTACAGACTTTGGCAGTCTTATTCCACACTGTCGCGCTACCATACTGCATCGGTCTGTTCTTTCTCATGCTCGCGTTCCCTCCTTTTTGGCCATTATTGGTAATGTATGTCATATACGCATACGGGTTCGACCACTCGAGCTCGAACGGAGAGATCTCCCGCCGGCGATCGCCGCTGTTTCGAAGACTCCCGTTGTTCAGGCTGTATTGTGATTACTTCCCCATCCACATTCACCGGGAGGTTCCGCTCGAGCCGACGTTTCCTGGTCGCCTTCGCGAACCGAGTGGCCTTGTCGAGCGGTGGATTGCGAAGATGTTCGGCGTGCAGGACGCTGTTGTCGAGGGAAATGAATCTGACGTTAAGGCCACGGCCAACGGCAATGGGACGACGAAAGAAATCGGACCGACGTATGTTTTCGGCTATCATCCGCATGGAATTGTTAGCTTGGGTGCGTTTGGTGCTATTGGTACGGAAGGCGCTGGATGGGAGAAGCTCTTTCCTGGGATCCCGGTGTCACTGCTGACTCTCGAAACAAATTTCAGCCTTCCATTTTACAGAGAGTATTTGCTGTCACTTGGGATTGCTTCAGTATCTCGACGGTCTTGTACCAATCTCCTCAAACACGACCAATCCATCTGCATCGTTATCGGCGGCGCCCAAGAGTCGCTCTTAGCGGAACCAGGCACTCTAGATCTGATCCTCGTTAAACGTCGCGGTTTTGTCAAACTTGCAATGTCAACGGCGCGGGTATCTGACCAACCGATTTGTCTTGTTCCGATCCTCAGTTTCGGCGAGAACGACGTGTACGACCAAGTCCGCGGGGACCGATCGTCGAAGTTGTATAAGATCCAGACTTTTATCAAGAAAGCGGCCGGGTTTACGCTACCATTGATGTATGCGCGCGGTATATTTAATTACGACTTTGGGCTGATGCCGTACCGCAGGCAAATGACGCTCGTGGTCGGCAAGCCGATTGCAGTGCCGTACGTGGCCCAGCCTACGGAGGCTGAAATCGAAGTGTATCACAAGCAGTACATGGATGAATTGAGGAGGTTATGGGACACGTATAAGGACGACTATTTTGTAGACCACAAGGGCAAGGGGGTCAAGAATTCCGAGATGCGTTTTGTGGAGTAA |
| NG69 | ATGTCCGAGAAGGCTGAGATTGAGGTGCCCCCCCAGAAGTCTACTTTCCCTCGATCCGTTCATTTCGCCCCCCTGCATATCCCCCTGGAGCGACGACTCCAGACCCTGGCTGTGCTCTTCCACACTGTTGCCCTGCCTTACTGCATCGGACTCTTCTTTCTGATGCTCGCTTTCCCCCCTTTTTGGCCCCTGCTCGTGATGTACGTTATCTACGCCTACGGATTCGACCATTCCTCTTCGAACGGCGAGATCTCTCGACGACGATCGCCTCTGTTCCGACGACTGCCCCTCTTTCGACTCTACTGTGATTACTTCCCTATCCACATTCATCGAGAGGTCCCCCTGGAGCCTACCTTTCCTGGTCGACTGCGAGAGCCTTCCGGACTCGTTGAGCGATGGATTGCTAAGATGTTCGGTGTCCAGGACGCCGTCGTGGAGGGAAACGAGTCTGATGTGAAGGCCACCGCTAACGGAAACGGCACCACTAAGGAGATCGGCCCTACTTACGTCTTCGGATACCACCCCCATGGCATTGTGTCCCTGGGAGCCTTTGGCGCTATCGGTACCGAGGGTGCTGGATGGGAGAAGCTCTTCCCTGGTATTCCCGTCTCGCTGCTCACCCTGGAGACTAACTTCTCCCTCCCCTTTTACCGAGAGTACCTGCTCTCTCTGGGAATCGCCTCGGTGTCCCGACGATCGTGCACCAACCTGCTCAAGCACGACCAGTCTATCTGTATTGTTATCGGAGGTGCTCAGGAGTCCCTGCTCGCTGAGCCTGGAACCCTGGACCTCATTCTGGTCAAGCGACGAGGCTTCGTGAAGCTGGCCATGTCCACTGCTCGAGTGTCTGATCAGCCTATTTGCCTGGTTCCCATCCTCTCTTTCGGCGAGAACGACGTTTACGATCAGGTCCGAGGTGACCGATCCTCTAAGCTGTACAAGATTCAGACCTTCATCAAGAAGGCCGCTGGCTTTACTCTCCCTCTGATGTACGCCCGAGGCATCTTCAACTACGACTTTGGTCTGATGCCCTACCGACGACAGATGACCCTCGTTGTCGGCAAGCCTATTGCCGTCCCCTACGTGGCTCAGCCCACTGAGGCCGAGATCGAGGTCTACCACAAGCAGTACATGGACGAGCTGCGACGACTCTGGGATACCTACAAGGACGATTACTTCGTTGACCATAAGGGCAAGGGTGTCAAGAACTCTGAGATGCGATTTGTGGAGTAA |
| NG70 | ATGCCCCGAAACACCCACCCCCCCGCCAACAACGCCGGACCTAACGCCTCTCACAAGAAGGACCGAAAGCGACAGGGACGACTCTTTCAGCACACCGTTCCTAACAAGTACTCTCGAATCCGATGGGCCCCCCTCAACATTGGCCTGGAGCGACGACTGCAGACCCTCGTCGTGCTGTGCCATACCCTCACTATCGCCCTGTTCCTCGCTTTCTTTTTCTTTACTTGTGCCATTCCCCTGACCTGGCCTCTGCTCTTCCCCTACCTCGTGTACATCACCCTGTTTTCGACCGCTCCTACTTCCGGTACCCTGAAGGGACGATCTGACTTCCTCCGATCGCTGCCTATTTGGAAGCTCTACACTGCCTACTTTCCCGCTAAGCTGCACCGATCCGAGCCTCTGCTCCCTACCCGAAAGTACATCTTCGGCTACCACCCCCATGGTATCATTTCCCATGGAGCCTTCGCCGCTTTTGCCACTGACGCTCTCGGCTTCTCTAAGCTGTTTCCTGGTATCACCAACACTCTGCTCACCCTGGATTCGAACTTCCGAATTCCCTTTTACCGAGAGTACGCCATGGCTATGGGAGTGGCTTCCGTTTCTCGAGAGTCGTGCGAGAACCTGCTCACTAAGGGAGGTGCTGACGGAGAGGGAATGGGCCGAGCTATCACCATTGTTGTCGGAGGCGCCCGAGAGTCCCTCGATGCTCTGCCTCACACTATGCGACTGGTCCTCAAGCGACGAAAGGGTTTCATCAAGCTGGCCATTCGAACCGGAGCTGACCTCGTTCCCGTCCTGGCCTTCGGCGAGAACGACCTCTACGAGCAGGTGCGATCTGATCAGCACCCTCTGATCTACAAGGTCCAGATGCTCGTGAAGCGATTCCTGGGTTTTACCGTGCCCCTGTTCCATGCTCGAGGAATTTTTAACTACGACGTTGGCCTCATGCCTTACCGACGACCCCTGAACATCGTGGTTGGTCGACCCATTCAGGTCGTGCGACAGCAGGACCGAGATAAGATCGACGATGAGTACATTGACCGACTCCACGCCGAGTACGTCCGAGAGCTCGAGTCCCTGTGGGACCAGTGGAAGGATGTTTACGCCAAGGACCGAATCTCTGAGCTGGAGATTGTCGCTTAA |
| NG71 | ATGGCTGCTGTTCAGGTTGCCCGACCCGTTCCCCCCCACCACCACGATGGCGCTGGCCGAGAGCACAAGGGAGAGCGAGCCCATTCCCCTGAGCGAGGAGAGAAGACCGTCCACAACGGCTACGGTCTGGCCGAGACTCATGAGCCCCTGGAGCTCAACGGTTCTGCTGTGCAGGACGGAAAGCACGACTCGGATGAGACCATCACTAACGGTGACTACTCTCCCTACCCTGAGCTCGATTGCGGAAAGGAGCGAGCCGCTCATGAGAAGGAGGCTTGGACCGCTGGAGGTGTGCGATTCGCTCCTCTGCGAGTTCCTTTTAAGCGACGAATGCAGACTGCCGCTGTCCTCTTCCACTGCATGTCCATCATTCTGATTTCCTCTTGTTTCTGGTTTTCTCTCGCCAACCCCATCACCTGGCCTATTCTCGTTCCCTACCTGGTCCACCTGTCGCTCTCCAACGCTTCTACTGACGGCAAGCTCTCCTACCGATCTGAGTGGCTGCGATCCCTGCCTCTCTGGCGACTGTTCGCCGGTTACTTTCCCGCTAAGCTCCACAAGACCTTCGATCTGCCCCCTAACCGAAAGTACATCTTTGGTTACCACCCCCATGGAATCATTTCCCATGGCGCCTGGTGTGCCTTCGCTACCAACGCTCTGGGCTTCGTTGAGAAGTTTCCTGGTATTACCAACTCGCTGCTCACTCTCGACTCCAACTTCCGAGTGCCCTTTTACCGAGATTGGATCCTGGCCATGGGCATTCGATCTGTTTCGCGAGAGTCTATCCGAAACATTCTCTCGAAGGGAGGACCTGACTCCAACGGACAGGGCCGAGCTGTGACCATCGTTATTGGTGGAGCCCGAGAGTCTCTGGAGGCTCAGCCCGGAACTCTGCGACTCATTCTGCAGGGCCGAAAGGGCTTCATTAAGGTGGCTCTCCGAGCTGGAGCTGACCTGGTTCCCGTCATCGGTTTCGGAGAGAACGACCTCTACGATCAGCTGTCCCCTAAGACCCACCCCCTCGTTCATAAGATCCAGATGTTCTTTCTGAAGGTCTTCAAGTTTACTATTCCTGCTCTGCACGGACGAGGTCTGCTCAACTACGACGTCGGTCTGCTCCCTTACCGACGAGCTGTGAACATCGTCGTGGGACGACCCATCCAGATTGACGAGACCTACGGCGAGCAGCCCCCTCAGGAGGTCATCGATCGATACCACGAGCTCTACGTCCAGGAGGTGGAGCGACTGTACGCCGCTTACAAGGAGCAGTTCTCGAACGGAAAGAAGACCCCCGAGCTCCAGATCCTGTCCTAA |
| NG72 | ATGCTCGCCTGGATGCCTGTCCTCATTGCCCTCCCCCGACGAAAGCAGACCGCTGTTGTTCTCCTGTTTGTGATGCTCCTCCCTATGATCATGGTCGTGTACTCCTGGACCCTGATCCTGCTCATTTTCCCCCTCACCACTCTGCCTACTCTCTCCTACCTGATCTGGATTATGTACATTGACAAGTCTCACGAGACCGGAAAGCGAAAGCCCTTTATGCGATACTGGAAGATGTGGCGACATTTCGCCAACTACTTTCCTCTCCGACTGATCCGAACCACTCCCCTGGACCCTCGACGAAAGTACGTGTTCTGCTACCACCCCCATGGCATCATTTCCCTCGGAGCCTTCGGCAACTTTGCTACCGACTCGACTGGCTTCTCCCGAAAGTTTCCCGGTATCGATCTGCGACTGCTCACCCTCCAGATTAACTTCTACTGTCCTATCATTCGAGAGCTGCTCCTGTACATGGGTCTGTGCTCTGCCGCTAAGAAGTCGTGTAACCAGATCCTCCAGCGAGGACCCGGCTCTGCTATTATGCTGGTTGTCGGCGGTGCCGCTGAGTCCCTCGACTCTCAGCCTGGCACCTACCGACTCACTCTGGGTCGAAAGGGATTCGTGCGAGTTGCCCTGGACAACGGTGCTGATCTGGTCCCCGTGCTCGGTTTCGGAGAGAACGACGTGTTTGATACCGTTTACCTGCCCCCTAACTCGTGGGCCCGAAACGTCCAGGAGTTCGTGCGAAAGAAGCTCGGATTCGCTACCCCCATCTTTTCCGGCCGAGGTATTTTTCAGTACAACATGGGTCTGATGCCCCACCGAAAGCCTATCATTGTGGTTGTCGGAAAGCCCATCAAGATTCCCAAGATCCCTGACGAGCTGAAGGGACGAGCCCTCTCTACCACTGCCGAGGGCGTTGCTCTGGTCGATAAGTACCATGAGAAGTACGTTCGAGCCCTCCGAGAGCTGTGGAACCTCTACAAGGAGGAGTACGCTACCGAGCCCAAGGCCGCTTACCTCGAGCCTAACTCGATTCGAAAGAACCAGAACGTCTAA |
| NG16 | ATGGAAGTCCGACGACGAAAAATCGACGTGCTCAAGGCCCAGAAAAACGGCTACGAATCGGGCCCACCATCTCGACAATCGTCGCAGCCCTCCTCAAGAGCATCGTCCAGAACCCGCAACAAACACTCCTCGTCCACCCTGTCGCTCAGCGGACTGACCATGAAAGTCCAGAAGAAACCTGCGGGACCCCCGGCGAACTCCAAAACGCCATTCCTACACATCAAGCCCGTGCACACGTGCTGCTCCACATCAATGCTTTCGCGCGATTATGACGGCTCCAACCCCAGCTTCAAGGGCTTCAAAAACATCGGCATGATCATTCTCATTGTGGGAAATCTACGGCTCGCATTCGAAAACTACCTCAAATACGGCATTTCCAACCCGTTCTTCGACCCCAAAATTACTCCTTCCGAGTGGCAGCTCTCAGGCTTGCTCATAGTCGTGGCCTACGCACATATCCTCATGGCCTACGCTATTGAGAGCGCTGCCAAGCTGCTGTTCCTCTCTAGCAAACACCACTACATGGCCGTGGGGCTTCTGCATACCATGAACACTTTGTCGTCCATCTCGTTGCTGTCCTACGTCGTCTACTACTACCTGCCCAACCCCGTGGCAGGCACAATAGTCGAGTTTGTGGCCGTTATTCTGTCTCTCAAACTCGCCTCATACGCCCTCACTAACTCGGATCTCCGAAAAGCCGCAATTCATGCCCAGAAGCTCGACAAGACGCAAGACGATAACGAAAAGGAATCCACCTCGTCTTCCTCTTCTTCAGATGACGCAGAGACTTTGGCAGACATTGACGTCATTCCTGCATACTACGCACAGCTGCCCTACCCCCAGAATGTGACGCTGTCGAACCTGCTGTACTTCTGGTTTGCTCCCACACTGGTCTACCAGCCCGTGTACCCCAAGACGGAGCGTATTCGACCCAAGCACGTGATCCGAAACCTGTTTGAGCTCGTCTCTCTGTGCATGCTTATTCAGTTTCTCATCTTCCAGTACGCCTACCCCATCATGCAGTCGTGTCTGGCTCTGTTCTTCCAGCCCAAGCTCGATTATGCCAACATCTCCGAGCGCCTCATGAAGTTGGCCTCCGTGTCTATGATGGTCTGGCTCATTGGATTCTACGCTTTCTTCCAGAACGGTCTCAATCTTATTGCCGAGCTCACCTGTTTTGGAAACAGAACCTTCTACCAGCAGTGGTGGAATTCCCGCTCCATTGGCCAGTACTGGACTCTATGGAACAAGCCAGTCAACCAGTACTTTAGACACCACGTCTACGTGCCTCTTCTCGCTCGGGGCATGTCGCGGTTCAATGCGTCGGTGGTGGTTTTCTTTTTCTCCGCCGTCATCCATGAACTGCTTGTCGGCATCCCCACTCACAACATCATCGGAGCCGCCTTCTTCGGCATGATGTCGCAGGTGCCTCTGATCATGGCTACTGAGAACCTTCAGCATATTAACTCCTCTCTGGGCCCCTTCCTTGGCAACTGTGCATTCTGGTTCACCTTTTTCCTGGGACAACCCACTTGTGCATTCCTTTATTATCTGGCTTACAACTACAAGCAGAACCAGTAG |
| NG109 | atgacggagcgatcccttccagtgacgctccctcttcctcgaaactttgcgctcacaccgcaccagatggcctcgccagacccgccactcccaggcccagccaacctcgtcgacgacgcactccgacacccagactcggcgccgcccatctcgcccgactccgcgcctccttcgactgcgactcggccctctgctctctcgcgcggagagctctcgaccgcttcgagctacgcgagcgaggtgtcgacgagggaggggacaccggatctggcgaatgggcaaggggttacgacgaccatcacgactgtcacaggcaaaggcggaaaggccgtcacccagaccctcacccacgtcggcgccgcctccgtcgacgcccgcttctcctccaccacaaactccatcactctccgccctatccccgcccgtggcggcgacccgaaaaagatcaaagtcctccgctctcgtcggacccacttcgccccacgcacctcacacttcgaccgtcacaacctcacctccgcctctgacccgttccgcggactgtacacgttgttctggatcgtgatcttcgttggggcactcaagactgtgtatcatcggtttgcggaacagggtgggtggggtggagaatggaggtttgcggcgttgattagtcgcgatgggtgggttctggcggttagtgatgcggtgttggttagcgcgtcgttgttgtgcgtgccgtatgcaaagctcctcgtacacggctggatccggtaccacggcgcaggcgtcatcatccaacacatctgtcaaacgctctacctcgccatcgcgatccgctggaccttccaccgcaactggccctgggtccaaagcggtttcatgaccctccacgccctctcgatgctcatgaagatccatagctactgttctctgaacggcgagctttcggagcggcggagacagttgaagaaggacgagaagcggttggaggaggtgctggaggagatgggtggacggaggaaggcggagagggaggcgagggaggagtgggagaggcagtgtggggaggcggcgagggccaaggagggtgaggcgggagtgagcgagggggagaaggaggcggcggcgactctatcttcgacggatgcgtcgaattcggccctttcgtcggaggacgaggcggctgcggcgctgttgcggcatcgacagccgactgctcgacgacgatccatctcgccctctgcctcacgcaccggttcctcctccgccccctccgctaccctcgccccctctcgcgccgaagaaccccaagaaggcgttgagacgctcacctggcacccatccgaccaagtcagcaaactcgctatcgccatctgcgaggcaaaggacctcctcacgagtaacggcaagaagcccgtcacgttccccgagaacgtcacctttgcgaactttatcgactacttgcttgtgccgacgttggtgtacgagttggagtaccctcggacggattccatccggcccctctacatcctcgaaaagaccctcgcaaccttcggcaccttctccattctcgtcctcatcgtcgactcgttcatcctccccgtcacctcgcgcaccgacacgcccctcttcgggttcgtcctcgacctcgccctgccgttcacgctcgcgtacctcctcatcttctacgtcatctttgagggcgtgtgcaatgggtttgcggagttgacgaggtttgcggatcggaatttcttcgacgattggtggaactcgtgcacgttcgacgagttctcgcgcaagtggaatcgccccgtccacgccttcctcctccgccacgtttacgccgaaacgatggcttcttacaagctctcgaagctctcggctgcgttcgtcacgttcttgttcagcgcctgcgtgcacgaactcgtcatggcggtcgtgacgaagaagcttcggctgtacctgttctcgatgcagatggcccagctcccgctcatcatggtgggccgcgccaagatcttccgacagtatccagcgctcggcaacctcttcttctggctcgcccttctctcgggattcccgcttctcgggacgctgtatctgcggtactga |
| NG110 | ATGTCGACCGCTGCACAATCTGATACAGACAACGAGGATATATCGACTGTCGATTTGGTTGACTCTCGTGCAGATACTCACACATCTTCAAATGTTATGTTGCAACAGCAAAAATCGCGTCGGAGACTAATCGGGAAAGACGCCGAGCCAAGAACACAGCATCCGTCTGGAGGCAAATCGGAGAAGGAGGAGTTGACGAAGCCGGATGACTCAAAGGGACCCATAAAATTAAGTCACATATACCCGATACATGCCGTTAGCCGAGGCAGTATTCTGTCACGAGAGTCGACAACTCCTACACCGAGTTTTGTTGGGTTTCGAAACTTAGCCATGATAGTGCTAGGGAAGTTACAGTATTCATTATTCTTTTGGTGCGATCGGGCTAACATTCCGACAGCCGTCAGCAATCTTCGATTGGTGATTGAAAATTACTCAAAGTACGGCGTTCTGATCCGATTCGCCCGACTCGGTATTTCACAAAAGGACATTCTGTATTGCATATTCTTGACCGCTACCATCCCGCTGCACCTATTTATTGCTATTGTCATTGAAAGACTAGTTGCGATTCCGACGGTAAACTACGTCGCTTCGCTCAGCGAGAGCGAGGATAAAAAACGCTCCAACCCCAAAATGGGACGGAAGGGGGGCAGTATATCGATTTTGCGTCCTAAGCCAAAATATATGTGGCGCCTGATCGTCCTATTGCATTCAATAAACGCAATGGCTTGCTTGTGGGTTACGACTGTTGTTGTTTACAATTCTATTTATCATCCCCTTATTGGGACAGCTTGTGAATTTCATGCAGTGATTGTGTGTCTTAAGGTCGCATCGTTTGCGCTTACCAATCGCGATCTTCGGGAGTCGATGCTGAACTCTCAACCTGTGCCAGCCATATACAACTTGGCCCCTTATCCAAAAAACTTAACCCTCAAGAACTTGTCATACTTTTGGTGGGCGCCGACTCTTGTTTATCAACCTGTCTATCCGCGATCGCCTTCATTCCGGCCTTTGTTTTTTGTCAAGCGGATTCTGGAGATGGTGGGCCTATCATTTTTAATATGGTTCTTGTCAGCTCAATATGCTGTGCCGACGCTAGAAAATAGTTTGGTGCATTTTCACAGTTTGCAATTCATGGGAATTATGGAGCGACTCATGAAGCTTGCTAGCATTAGCATGGCTATTTGGCTTGCTGGTTTTTTCTGCATTTTTCAGTCTGGACTCAATGCGCTTGCGGAGGTAATGCGGTTTGGTGACAGAGCCTTTTACGACGACTGGTGGAACAGCAAATCTGTGGGAGAGTATTGGCGTCTGTGGAATAAGCCGGTTACGAATTACTTCCGGCGTCATATTTACGTACCGCTTGTGCGCCGCGGGTGGAATTCTGCGACAGCCAGTGTCATGGTATTTTTCGTCAGCGCGGTGTTGCATGAGCTAGTTGTTGGAGTTCCGACGCATAACGTAATTGGAGTTGCATTCTCGTCGATGATTCTACAAATCCCACTCATACAAGTAACCGCGCCTCTGGAGAAGATGCATGGACCTACATCTGGAATAATAGGGAACTGTATCTTTTGGTTTAGCTTCTTCATCGGTCAGCCTCTGGGCGTGCTACTTTACTATTTTGCGTGGAACGTTAGTATGAGCAAAGTAAAGATGGTCGAGAGCTAG |
| NG111 | ATGGTGATGGACACACAAACCACAGCATCCGCCACCAGCACGGCGCTCACGACCGACCACACTGTTGCCTCTCGGACGTCCCGCTCTGAGCCGAACGGTGGTGTGCATAATGTATCGTCACCTCCAACGAGCGAACCGACTGGGGGAAATGGCGGAGGCCGGCGAAGGAGTAAATACCGGCATGTCGCAGCGTACCATTCCGAAGTGCGCCATTCCAGTCTCAGTCGGGAATCGAATACTTCTCCGAGTTTCCTCGGATTCCGGAACCTCATGGTAATCGTATTAGGTGAGTGCCCTAGTGCTCTCCTACGTTTTGTGAACCCGACGGAGAACTCATACGGGTCGCGACTAGTTGCTATGAATCTTCGATTGGTTATCGAGAATTACGTGAAGTATGGGGTCTTGATCTGCATCAGATGCCACGATTATCGAAAGCAGGACGTTGTCCTGGGCTCAATGTTATTTGCTCTCGTCCCATGCCAGCTATTCATCGCCTACCTCCTGGAATTGGCCGCAGCGGGTAGGGCCAAACAGACTGTGGGCCGAAAGAAAAAGGACGGATCAGCCGAGGAGGGCGAACGTGAAGCACGTGCTTTTCGACACATCTGGCGGTTTGCATTGTCCTTTCACATCCTCAACATTGTTCTCAATCTCGCCGTCACGAGCTTCGTTGTGTATTACTACATCCACCATCCCGGCATTGGTACGCTCTGTGAAGTGCATGCGATCGTTGTCGCGTTGAAAAACTGGTCCTATGCGTTCACCAATCGGGATCTGCGAGAGGCGATGCTTAATCCCTCGGCGGAGTCGGCGCTTCCCGAGATCTATTCCAGCCTCCCGTACCCGAAAAACATCACGTTAGGAAATCTAACGTACTTCTGGCTTGCACCGACACTGTTGTATCAGCCAGTATACCCCAGGTCGCCTTCCATCCGATGGCCATTCGTGGCCAAACGCTTGTCGGAATTTGCGTGCTTGTCGGTGTTCATTTGGCTACTTTCGGCCCAATACGCTGCGCCAGTTTTGCGCAACTCCATTGACAAGATTCGTGATATGGCATATGCATCCATTTTTGAGCGCGTTATGAAGCTATCCACCATCTCTCTCGTCATTTGGCTGGCTGGGTTCTTTGCGATTTTCCAATCACTCTTGAATGCTTTGGCGGAGATCATGAAGTTTGGCGATCGGGAATTCTACACCGATTGGTGGAATAGCCCAAGTCTCGGTGTTTACTGGCGGTCATGGAATCGGCCAGTGTACCAGTTCATGAAGCGGCACGTATATTCTCCGTTGATAGGGCGGGGGTACAGCCCGTTTGTGGCAAGCACTGTCGTATTCACCATCTCCGCTCTCCTTCATGAGCTCCTCGTGGGGATACCCACGCACAACATGATAGGCGTCGCGCTTGTTGGAATGCTGTTCCAGCTCCCGTTGATCGCCATCACTGCCCCATTGGAAAAGATGAAAGATCCATTGGGTAAGCCCCTGGGAGCACTGCTGTATTTCTTTGCCTGGCAGGCAAAATATGGCAGTGTGAGCAGGATGGGCAACTGA |
| NG112 | atgtccgccacgggcgttgatgtggccaacggccgcagcggcgcgcgacgacgcaacgatactgccgtcgacgagactatatccgccgtcacggccgagatgcgttcctcgtcgcatccaacataccgccatgtgtctgctgtgcactccacgagccggccctcgtgtctgagccatgattctgacgctgcgccgagcttcattggctttcgaaatctcatggtcattgttctggtcgttggcaatgttcgattaatgattgaaaatctaaaaaagtacggcgtactgatatgcctccgatgtcactcgtataaaaacgaagacatcattatcggcggactgctctacttcctgatcccctgccacttgcttgtcgcctacggaatcgagttagccgccgccagacaagcacgcgaatctcgaactcgtccaccaggccagtccgacacggcgtcgaaatcaacagaagatgacaacaagcacttccactcaacatgggtgctcgctgcctgggcacacatcatcaacatgacactttccttcatcctcaccaccttcgtcgtctactactacgtgcaccatcccctcgtcggcaccctgaccgagatgcacgccgtcatcgtctctctcaaaacagcttcctacgcattcaccaaccgagatcttcgccacgcatacctccatcctgacaagcgcaagcacatccccgagctatatctcgaatgtccctacccccagaacctcacctttggcaatctcgtgtatttctggtgggcccccacgctggtataccagcccgtgtatccgcgcaccgacaagatcagatgggtttttgtttttaaaagactaggcgaagtctgctgtctcagcgcattcatctggttcgccagcttccagtacgccgcgcccgtgttgcggaactccctggacaagattgcgtctctcgacttcatcatgatctttgagcgccttctcaagctatccaccatttctctcgtcatctggctcgccggcttcttcgccctgttccagtctttcctgaatgccctggctgaggtattgcgctttggggaccggtgcttctacgacgattggtggaatagcgagagtctgggggcgtattggaggacgtggaacaggcctgtgtatacctacttcaagcgccatgtgtatgtgcccatgattgggaggggatggagtccctggactgctagttgtactgttttttttgtgtcggcggtgctgcacgaggttcttgttggggtgcccacccacaatatcattggtgtcgcctttgtgggcatgtttctgcagcttcccctaatagccctcaccgctcccatggaaaagaagaaatggggccacaccggccgtgtgatgggcaatgttattttctgggtgtcctttacaatctttgggcagccctttgcagcgctcatgtacttttatgcctggcaggccaagtacgggagcgtgagtcggcaaattgtgctggtgaatccggtggaggaggcgtcttga |
| NG113 | atgaaggcagaaacgggcacaacgatggcaacgtcgactagtctcgagacttcccaagtcaatggcgtcaccaaccgggcccctgttggccctagtcacgacccccacgctacaactccgactcatgagacgacaaccaccataccgtccgacgtcctcgccaatggttctacaaatgggactacgaatgggacgacagatgattcattggacatatccgaattgcgcaaagcgttccgcaacaagtatcgccatgtcgaggctgtccactccgaatcgaaaccatcctgtctgagccatgacgctacagagacacccagtttcatcggttttaggaatctcatggtgattgtgttggttgctgccaatcttcgcctggtcatcgagaacattcaaaagtatggagttctgatctgcatcaaatgccacgactttcgccccaacgatgtacgcctggggctcctcctctacatcctgatcccatggcacctcatgctcgcctacctcattgagctggtcgccgccgccaatgcccgcaactcccgggccaaggcgaagaagcgggacggcagtaccagcccgaccgaagacgagtccaagcaattcctgcagacctggcggatgctccgcattctccacgccgtcaacgtcacggccgccctggccgtcacctcctacgtggtctactactacattcaccacccgctgatcggcacgctctcggagctgcacgccatcatcgtgtggctcaagacggcgtcgtacgcgctcaccaaccgcgacctgcgccacgcctacctacacccggtgcgcggcgagcgcgacgctctgcccgagatctacgcccagtgcccctacccggccaacgtgaccttctccaacttgacctacttctggtgggcgcccaccctggtgtaccagccggcgtacccgcgcactcagcgcatccgctgggtctttgtggctaagcgcctcggcgaggtcgtctgcttgagcgccttcatctggttcgccagcgcccagtacgctacccccgtgctgcgaaactcgctcgacaagatcgctaccctggattacatgtccattgtcgagcgtctgttgaagctgtcgaccatctcgctggtcatctggctggcgggcttctttgcgctgtttcagagtttcctgaatgccttggccgaggtgatgcggtttggagaccgcgagttctacgaagcatggtggaacagcgaaagcctcggcgcctactggcgcacctggaacaaacccgtgtaccaattcttccggcggcacgtctactcgccgatgcggtcgcgcgggtggagccacttgtcggccagcctcgccgtgtttctgctctcggccgtgctacacgagctgctggtgggggtgccgacgcacaacatcatcggcgtcgccttcctgggcatgttcctgcagctgccgctcatcgccatgacggcgcgcctgggcggccgccgcgggaacaccgcccacggccgcctgctcggcaacactatcttttgggtgtcatttaccatttttggccagccgtttgccgcgctgatgtatttttatgcatggcaggccaagtatggtagtgtgagcaagatgccgctggcgcagccggggacgtgtccggctgtggttgtttga |
